# Differential Discontinuation Profiles between Pirfenidone and Nintedanib in Patients with Idiopathic Pulmonary Fibrosis

**DOI:** 10.3390/cells11010143

**Published:** 2022-01-02

**Authors:** Kazutaka Takehara, Yasuhiko Koga, Yoshimasa Hachisu, Mitsuyoshi Utsugi, Yuri Sawada, Yasuyuki Saito, Seishi Yoshimi, Masakiyo Yatomi, Yuki Shin, Ikuo Wakamatsu, Kazue Umetsu, Shunichi Kouno, Junichi Nakagawa, Noriaki Sunaga, Toshitaka Maeno, Takeshi Hisada

**Affiliations:** 1 Department of Respiratory Medicine, Gunma University Graduate School of Medicine, 3-39-15, Showa-machi, Maebashi 371-8511, Japan; k-kanplude3@jcom.home.ne.jp (K.T.); g.yr.328@gmail.com (Y.S.); m09702007@gunma-u.ac.jp (M.Y.); shiki0246@gmail.com (Y.S.); nsunaga@gunma-u.ac.jp (N.S.); mutoyu03@gunma-u.ac.jp (T.M.); 2 Department of Respiratory Medicine, Public Tomioka General Hospital, 2073-1, Tomioka 370-2393, Japan; 3 Department of Respiratory Medicine, Maebashi Red Cross Hospital, 389-1, Asakura-machi, Maebashi 371-0811, Japan; yhachisu2002@yahoo.co.jp; 4 Department of Respiratory Medicine, Kiryu Kosei General Hospital, 6-3, Orihime-machi, Kiryu 376-0024, Japan; mutsugi@gaea.ocn.ne.jp; 5 Department of Respiratory Medicine, Isesaki Municipal Hospital, Tsunatorihonchou 12-1, Isesaki 372-0817, Japan; sprq6fc9@road.ocn.ne.jp; 6 Department of Respiratory Medicine, Tone Central Hospital, 910-1, Numasu-machi, Numata 378-0012, Japan; s-yoshimi@msi.biglobe.ne.jp; 7 Department of Respiratory Medicine, National Hospital Organization Takasaki General Medical Center, 36, Takamatsu-cho, Takasaki 370-0829, Japan; i_18_waka@outlook.jp (I.W.); jnakaga@yahoo.co.jp (J.N.); 8 Department of Respiratory Medicine, Fujioka General Hospital, 813-1, Nakakurisu, Fujioka 375-8503, Japan; emuirakirak@gmail.com (K.U.); contra.since2005@gmail.com (S.K.); 9 Graduate School of Health Sciences, Gunma University, 3-39-22, Showa-machi, Maebashi 371-8514, Japan; hisadat@gunma-u.ac.jp

**Keywords:** idiopathic pulmonary fibrosis, pirfenidone, nintedanib, discontinuation, body mass index, adverse event, antifibrotic treatment

## Abstract

Antifibrotic agents have been widely used in patients with idiopathic pulmonary fibrosis (IPF). Long-term continuation of antifibrotic therapy is required for IPF treatment to prevent disease progression. However, antifibrotic treatment has considerable adverse events, and the continuation of treatment is uncertain in many cases. Therefore, we examined and compared the continuity of treatment between pirfenidone and nintedanib in patients with IPF. We retrospectively enrolled 261 consecutive IPF patients who received antifibrotic treatment from six core facilities in Gunma Prefecture from 2009 to 2018. Among them, 77 patients were excluded if the antifibrotic agent was switched or if the observation period was less than a year. In this study, 134 patients treated with pirfenidone and 50 treated with nintedanib were analyzed. There was no significant difference in patient background, discontinuation rate of antifibrotic treatment over time, and survival rate between the two groups. However, the discontinuation rate due to adverse events within one year of antifibrotic treatment was significantly higher in the nintedanib group than in the pirfenidone group (76% vs. 37%, *p* < 0.001). Furthermore, the discontinuation rate due to adverse events in nintedanib was higher than that of pirfenidone treatment throughout the observation period (70.6% vs. 31.2%, *p* = 0.016). The pirfenidone group tended to be discontinued due to acute exacerbation or transfer to another facility. The results of this study suggest that better management of adverse events with nintedanib leads to more continuous treatment that prevents disease progression and acute exacerbations, thus improving prognosis in patients with IPF.

## 1. Introduction

Idiopathic pulmonary fibrosis (IPF) is a chronic, progressive, and fatal lung disease of unknown etiology. Environmental and occupational exposures have been suggested to play a role in the pathophysiology of IPF [1,2]. Recently, we reported inhaled silica/silicates in the lungs to be associated with the progression and prognosis in patients with IPF [3]. As an antifibrotic treatment for IPF in Japan, nintedanib became available in 2015, in addition to pirfenidone, which was available since 2008. Pirfenidone has also been reported to be effective in treating familial IPF [4]. However, IPF has an average survival time of 3–5 years and the poorest prognosis among interstitial pneumonias [5].

Pirfenidone was the first approved oral antifibrotic drug. In the CAPACITY trial, pirfenidone decreased the decline in forced vital capacity (FVC) at 72 weeks after treatment [6]. The ASCEND trial evaluated that 52 weeks of treatment reduced the decrease in percent predicted FVC (%FVC) and improved progression-free survival at 52 weeks from baseline [7].

Nintedanib is a multi-intracellular tyrosine kinase inhibitor that targets vascular endothelial growth factor, platelet-derived growth factor, and fibroblast growth factor. The TOMORROW trial showed that compared with placebo, nintedanib treatment resulted in a reduced annual decline in FVC [8]. The INPULSIS trials showed similar favorable results and prolonged the time to the first acute exacerbation [9]. The INPULSIS-ON study showed a long-term safety and toxicity profile [10]. However, the continuity of nintedanib treatment in Asian populations is extremely poor, and its long-term treatment with nintedanib has become a critical issue [11,12,13]. There still are few reports comparing the drug-related adverse events of these two antifibrotic drugs.

Therefore, in this study, we compared the clinical background, effects, adverse events, and prognosis of IPF upon treatment with pirfenidone and nintedanib and examined their continuity and tolerability.

## 2. Materials and Methods

### 2.1. Study Population

**Figure 1:**
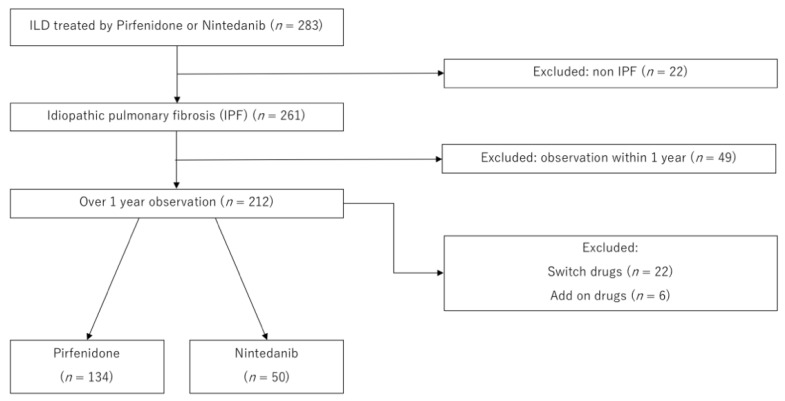
Study population.

We retrospectively recruited 283 patients with interstitial pneumonia (IP), including 261 who were treated with pirfenidone or nintedanib between January 2009 and December 2018 at six regional core facilities in Gunma prefecture, Japan (Figure 1). To ensure data accuracy, we enrolled patients with an observation period of one year or more. A total of 77 patients were excluded for the following reasons: 49, because the observation period was less than one year and 28 because they were treated with pirfenidone and nintedanib in combination or had switched the drugs. This study was conducted in accordance with the tenets of the Declaration of Helsinki and was approved by the Gunma University Hospital Institutional Review Board (approval number: 150021).

### 2.2. Diagnosis of IPF and Data Collection

The diagnosis of IPF was based on a multidisciplinary discussion according to the official statement of the American Thoracic Society, European Respiratory Society, Japanese Respiratory Society (JRS), and Latin American Thoracic Association [14]. The date of data collection was at treatment initiation. Discontinuation was defined as a permanent termination of antifibrotic treatment.

### 2.3. Disease Severity

Disease severity was assessed by the gender, age, and physiology (GAP) staging system or the JRS severity staging system. JRS severity was classified as per arterial partial pressure of oxygen (PaO_2_) and the 6-min walk test [15]. The stage was defined as stage I, if PaO_2_ was more than 80 Torr; stage II, if PaO_2_ was more than 70 Torr and not less than 80 Torr; stage III, if PaO_2_ was more than 60 Torr and not less than 70 Torr; stage IV, when less than 60 Torr. Stages II or III were changed to III or IV, respectively, if desaturation (< 60 Torr) was obtained during the 6-min walk test.

### 2.4. Statistical Analysis

Statistical analyses were performed using the statistical software “EZR” (easy R), which was based on the R and R commander [16,17]. Comparisons of categorical data between the pirfenidone and nintedanib groups were performed using Fisher’s exact test. Continuous variables were analyzed using the Mann–Whitney U test and denoted by the median (maximum and minimum) [18,19]. Survival time was analyzed using the Kaplan–Meier method and compared between groups using the log-rank test. Statistical significance was defined as *p* < 0.05.

## 3. Results

### 3.1. Clinical Characteristics, Laboratory and Physiological Data, and Prognosis

We enrolled 261 consecutive IPF patients treated with antifibrotic drugs, pirfenidone or nintedanib, between 2009 and 2018. Clinical characteristics and laboratory and physiological data of patients treated with pirfenidone or nintedanib are shown in Table 1.

The median age of patients at the initiation of treatment with pirfenidone or nintedanib was 71 (range, 43–90 years) or 72 (range, 39–87 years) years, respectively. Men accounted for 73.1% and 86.0% of the two treatment groups, respectively. The median %FVC were 74% and 70% and the percent predicted diffusing capacity of carbon monoxide (%DLCO) were 54.4%, and 51.4%, respectively. Serum albumin levels were significantly lower in the pirfenidone than in the nintedanib group (3.8 vs. 4.0 g/dL). The median treatment periods of pirfenidone and nintedanib were 387 and 351 days, respectively. The median annual decline in FVC was 0.100 and 0.180 L, respectively, albeit without statistical significance.

Initial doses of antifibrotic drugs were different between pirfenidone and nintedanib. Pirfenidone was initiated at a lower dose of 600 mg/day for the first 2 weeks; thereafter, doses were sustained, increased or terminated. Nintedanib was administered at a dose of 300 mg/day according to the Japan Pharmaceutical Reference, then sustained, or reduced by 200 mg/day or terminated. The mean final doses of pirfenidone and nintedanib were 1153 ± 420.6 mg and 249 ± 59.69 mg, respectively.

### 3.2. Discontinuation Rates over Time

**Figure 2:**
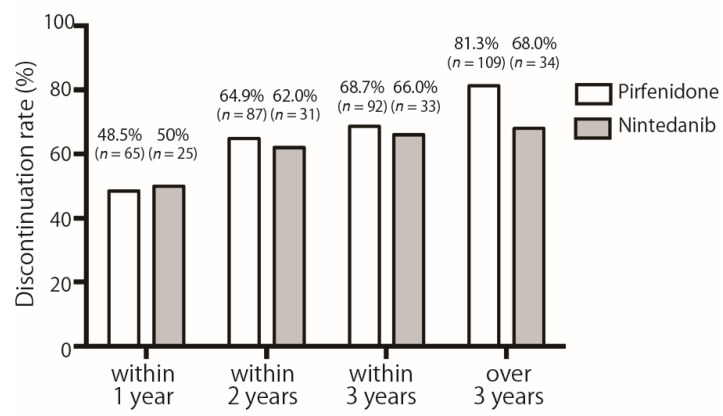
Discontinuation rate of pirfenidone and nintedanib over time.

Since we frequently experienced adverse events interrupting antifibrotic treatment, the rate of discontinuation of each agent within one, two, or three years was compared. Discontinuation rates within one year were 48.5% and 50.0% in the pirfenidone and nintedanib groups, respectively (Figure 2). There was no significant difference in the discontinuation rates between the two drugs throughout the study period. 

The discontinuation rate of both drugs within one year after initiation was approximately 50%. The increase in the interruption rate of both drugs over time was gradual over time. The number of cases (*n*) is the cumulative number.

### 3.3. Discontinuation Reasons

The reasons for the discontinuation of the two drugs were compared. Over the entire treatment period, the discontinuation rate due to adverse events was significantly higher in the nintedanib group than in the pirfenidone group (70.6 vs. 31.2%, *p* = 0.016). Compared with the pirfenidone group, diarrhea and liver dysfunction were the more common reasons for discontinuation in the nintedanib group (Table 2, Supplementary Figure S1).

**Figure 3:**
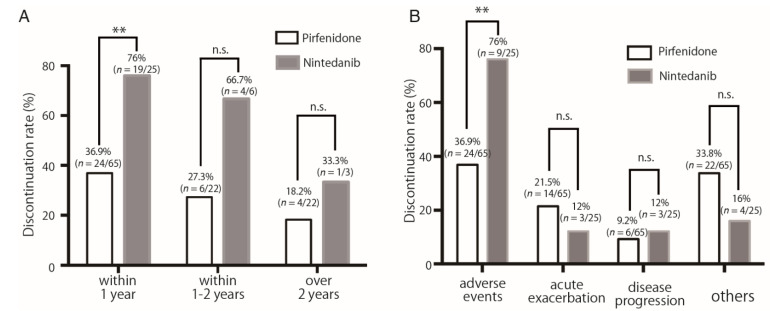
Comparison of (**A**) discontinuation rates due to adverse events over time and (**B**) discontinuation profiles within a year in the pirfenidone and nintedanib treatment. ** *p* < 0.01.

Next, the discontinuation rate due to adverse events over time was compared between the two groups. Discontinuation due to adverse events decreased over time in the pirfenidone and nintedanib groups, while the discontinuation rate due to adverse events exceeded 50% within one and 1–2 years in the nintedanib group (Figure 3A). Since half of the patients discontinued antifibrotic drugs within a year, we compared the reasons for discontinuation of both drugs within the first year. The discontinuation rate due to adverse events within the first year was significantly higher in the nintedanib group than in the pirfenidone group (76.9% vs. 36.9%, *p* = 0.001) (Figure 3A,B). However, there was no significant difference in the discontinuation rates between the two groups for other reasons, acute exacerbation, or disease progression (Table 2, Supplementary Figure S1). Interestingly, the pirfenidone group showed a decrease in the rate of adverse events resulting in discontinuation over time, while the nintedanib group showed a high rate of adverse events resulting in discontinuation even after 1 year of treatment. In addition, the rate of discontinuation due to adverse events was higher in the nintedanib group than in the pirfenidone group. Specifically, patients treated with nintedanib had experienced multiple adverse events, such as liver dysfunction and diarrhea, causing discontinuation.

(A) The adverse event discontinuation rate for pirfenidone was less than 50% from the first year, while nintedanib was still above 50% after the first year. The discontinuation rate of nintedanib due to adverse events in the first year was significantly higher than that of pirfenidone in the first year.
(B) Comparison of first-year causes leading to discontinuation of pirfenidone and nintedanib. A comparison of reasons for discontinuation in the first year showed that nintedanib had fewer acute exacerbations and significantly more adverse events than pirfenidone. ** *p* < 0.01.

### 3.4. Survival Time

**Figure 4:**
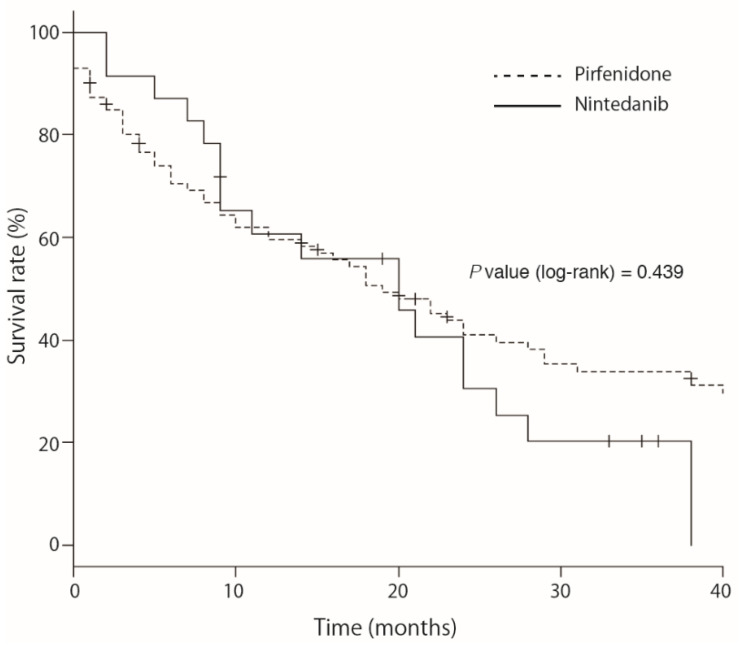
Kaplan-Meier survival analysis between pirfenidone and nintedanib treatment in patients with idiopathic pulmonary fibrosis.

The survival time after antifibrotic treatment is shown in Figure 4. The median survival times in the pirfenidone and nintedanib groups were 19 months and 20 months, respectively. Kaplan–Meier survival analysis showed that there was no significant difference in survival periods between the pirfenidone and nintedanib groups.

Kaplan-Meier survival analysis did not show a significant difference between pirfenidone- and nintedanib-treated patients with idiopathic pulmonary fibrosis. (19 months for pirfenidone (95% confidence interval, 12–28) vs. 20 months for nintedanib (95% confidence interval, 9–26), respectively; *p* = 0.439). Chi-square tests of 1-year (*p* = 0.603) and 2-year (*p* = 0.611) survival rates also showed no significant difference between the pirfenidone and nintedanib groups.

## 4. Discussion

The rate of discontinuation due to adverse events was as high as 50% even after 1 year or more of nintedanib treatment, exceeding that of the pirfenidone treatment, throughout the observation period. Notably, there was no significant difference in the interruption rate of both drugs over time. To the best of our knowledge, this is the first study to compare the discontinuation profiles of the two key antifibrotic drugs, pirfenidone and nintedanib.

### 4.1. Discontinuation of Pirfenidone

In a post-marketing surveillance study including 1371 patients in Japan, only 48% of patients treated with pirfenidone had a longer therapy duration of over one year [20]. In our study, the most common reasons for discontinuation of pirfenidone were acute exacerbations, disease progression, or hospital transfers, caused by IPF progression. Prevention or interventions for these events are difficult in patients with IPF. Similarly, patients with advanced IPF were associated with the discontinuation of pirfenidone within one year [21,22].

### 4.2. Discontinuation of Nintedanib

In the INPULSIS study, the interruption rate due to adverse events was as low as 10%, and the same result was obtained with INPULSIS-ON [9,10]. However, reports limited to Asian populations showed that the discontinuation rate of nintedanib was approximately 50% [11]. According to a study limited to Japanese patients, 40% were forced to discontinue nintedanib at a regular dose (300 mg/day) within six months [23]. Another study demonstrated a similar rate of discontinuation of nintedanib [13]. A key insight from our study is that attention should be paid to the occurrence of adverse events not only at the beginning, but also during treatment with nintedanib.

The overall discontinuation rates for pirfenidone and nintedanib were 81.3% (*n* = 109/134) and 68% (*n* = 34/50), respectively. In the analysis of reasons for discontinuation, the overall discontinuation rates due to adverse events of pirfenidone and nintedanib were 25.3% (34/134) and 48% (*n* = 24/50), respectively. Furthermore, the overall discontinuation rate due to disease progression or hospital transfer was 27.5% (*n* = 30/134) for pirfenidone, while it was 8% (*n* = 4/50) for nintedanib. This appears to have resulted in a discrepancy in the difference between the overall discontinuation rate and the adverse event-related discontinuation rate between the two drugs.

### 4.3. Risk Factors of Nintedanib Adverse Events

In recent years, countermeasures against adverse events have been reported. Kato et al. reported that a low body mass index (BMI) of 21.6 or less was a risk factor for diarrhea in patients treated with nintedanib [24]. It has been reported that the use of two or more intestinal regulators aided longer treatment with nintedanib [25], while diarrhea caused by nintedanib is suppressed not only by the use of antidiarrheal medication [26] but by dose reduction as well [25]. Poor performance status tends to cause nausea with the annual decline in FVC being poor; thus, the average survival time is poor in patients with nausea symptoms [24]. Since the introduction of nintedanib at 300 mg/day is likely to cause nausea, a reduced starting dose of 200 mg/day was suggested for cases of poor performance status.

Liver dysfunction caused by nintedanib was more likely to occur in patients with low body surface area and BMI and is often ameliorated by the termination of nintedanib [27]. Approximately half of the patients continue nintedanib with liver dysfunction by dose reduction [12,27]. In the INPULSIS-ON trial, dose reduction due to adverse events did not affect the annual rate of FVC decline [10]. Therefore, it is important to reduce the dose, if necessary. Additionally, patients with low body surface area and BMI may also consider a 200 mg/day dose at the start of nintedanib administration. Regarding the start of dose reduction of nintedanib (200 mg/day) in Japanese patients, there was less early discontinuation, liver dysfunction, and gastrointestinal adverse events than the start of regular dose (300 mg/day), and there was no significant difference in the decrease in annual decline in FVC [23].

### 4.4. Discontinuation of Nintedanib in Asian Population

A subgroup analysis of the INPULSIS trial reported that Japanese patients had a higher frequency of adverse events leading to discontinuation than the overall population [28]. In the report, the BMI of Japanese patients was 24.4, despite the BMI of the overall population being 28.1, showing a tendency of lower BMI in Japanese patients. Ikeda et al. suggested that a relatively smaller physique was associated with an increased incidence of severe adverse events, causing termination of nintedanib treatment [27]. Kato et al. reported that patients with a median BMI of 22.8 treated with nintedanib had a one-year discontinuation rate of 51% [13]. Combining the insights of these studies with ours, there seem to be many reasons for discontinuing nintedanib treatment following adverse events, for which a lower BMI is considerably associated.

Antifibrotic drug therapy that considers personalized differences such as racial and physical, has aided the longer-term and continuous treatment, leading to better management of IPF. Since nintedanib is often discontinued due to adverse events, paying attention to adverse events such as diarrhea and liver dysfunction at the initiation of nintedanib treatment may lead to tolerability and longer antifibrotic therapy, thus improving prognosis.

### 4.5. Limitations

This study had several limitations. First, this was a retrospective design. Prospective studies are required to evaluate the significance of managing adverse events, thus reducing the discontinuation of antifibrotic treatment. Second, the number of patients included was small, and fewer patients were treated with nintedanib than with pirfenidone. However, the baseline characteristics in our study were similar to those reported in previous studies [11,13,21]. In this study, there was no difference in the antifibrotic effect on annual declining FVC, treatment duration, discontinuation rate over time, and median survival time between pirfenidone and nintedanib. These results were consistent with those of previous real-world studies showing similar effects on the decline of annual FVC or survival time [29,30,31]. Third, during the post-marketing surveillance period, all institutions registered unified adverse events; however, one of the limitations of this study is that the subsequent registration of adverse events was entrusted to the medical record description at the discretion of the attending physician. It is necessary to unify them and examine the effect of side-effects prospectively, for better management. Fourth, the reasons for discontinuation of the antifibrotic treatment varied. The transfer was due to the progression of the disease in this study. IPF end-of-life care has many problems as it is not covered by hospice treatment health insurances such as cancer end-of-life care. In Japan, most IPF patients die in regional core hospitals, but some IPF patients are transferred from core hospitals to non-acute hospitals and meet their demise. According to a study on end-of-life care for IPF in Japan, hospice mortality rates were 36.4% and 0.6% for lung cancer and IPF, respectively [32]. In our study, antifibrotic treatment was discontinued due to transfer to another hospital as expensive nintedanib-based treatments were not covered by Japanese health insurance at the hospital where the terminal treatment of IPF was performed. Therefore, in Japan, after transfer from a core hospital to a non-acute hospital, patients may obtain informed consent and discontinue antifibrotic treatment. Furthermore, acute exacerbations were considered a reason for discontinuation apart from other adverse events. Acute exacerbations are a fatal, unlike other adverse events. Several studies have also focused on the incidence of acute exacerbations during antifibrotic treatment, and our study also analyzed the incidence of acute exacerbations, distinct from other adverse events. Case fatality rates for acute exacerbations were 18/34 (52.9%) and 3/12 (25%) (*p* = 0.356) in the pirfenidone and nintedanib groups, respectively. IPF treatment may be discontinued for various reasons and is a clinical issue for future IPF treatment.

## 5. Conclusions

There was no significant difference in the effect on annual FVC reduction, duration of oral administration, discontinuation rate, and median survival time between pirfenidone and nintedanib treatment. Treatment with nintedanib was frequently discontinued because of adverse events during the treatment period, unlike pirfenidone, which is often discontinued due to progression of IPF or acute exacerbations. Paying attention to the initial dose of nintedanib adjusted to the differences in physique and careful management of adverse events throughout the treatment may contribute to the longer nintedanib treatment with superior prognostic effects in patients with IPF.

## Figures and Tables

**Table 1:** Background and treatment of all patients.

Factor	All Patients (*n* = 184)	Pirfenidone (*n* = 134)	Nintedanib (*n* = 50)	*p* Value
**Characteristics**				
Age (years)	71 (39–90)	71 (43–90)	72 (39–87)	0.237
Male (male/female)	141 (76.6)	98/36 (73.1%)	43/7 (86%)	0.079
Body mass index (kg/m^2^)	22.6 (13.7–36.7)	22.3 (13.7–36.7)	23.1 (14.3–28.1)	0.307
Body surface area (DuBois, m^2^)	1.65 (1.18–2.58)	1.63 (1.18–2.16)	1.66 (1.23–2.58)	0.232
**Physiologic Marker before Treatment**				
FVC (L)	2.20 (0.62–4.56)	2.17 (0.62–4.56)	2.31 (1.03–4.16)	0.838
%FVC (%)	71.8 (27.4–124.9)	74.0 (27.4–124.9)	70.0 (31.0–116.5)	0.761
%DLCO (%)	52.6 (31.0–137.4)	54.4 (3.1–137.4)	51.4 (17.1–76.7)	0.138
JRS severity grade (I/II/III/IV/unknown)	23/12/63/61//25	20/7/40/46//21	3/5/23/15//4	0.104
GAP staging system (−2~0/1/2/3)	6/8/26/29	6/5/20/21	0/3/6/8	
(4/5/6/7/8//unknown)	28/19/6/4/1//57	20/12/3/3/0//44	8/7/3/1/1//13	0.624
**Serological Marker**				
Albumin (g/dL)	3.9 (2.1–4.7)	3.8 (2.1–4.6)	4.0 (2.6–4.7)	0.018 *
CRP (mg/dL)	0.26 (0.00–21.87)	0.29 (0.00–21.87)	0.25 (0.03–2.67)	0.280
KL-6 (U/mL)	1260.0 (223.0–9370.0)	1253.0 (303.0–9370.0)	1305.0 (223.0–8593.0)	0.762
SP-D (ng/mL)	237.0 (20.6–1100.0)	237.0 (29.5–1100.0)	259.5 (20.6–728.0)	0.544
**Treatment Period and Disease Progress**				
Final amount (mg)		1153 ± 420.6	249 ± 59.69	
Observation periods (days)	390 (2–2575)	389 (2–2575)	395 (5–1172)	0.758
Administration period (days)	378 (2–2575)	387 (2–2575)	351 (5–1172)	0.651
FVC decline per a year (L)	0.110 (−3.26–8.21)	0.100(−3.26–8.21)	0.180 (−0.70–1.26)	0.573

FVC, forced vital capacity; %FVC, % predicted forced vital capacity; %DLCO, % predicted diffusing capacity for carbon monoxide. Values are median (minimum-maximum) or number (percentage). The nominal variables were analyzed using Fisher’s exact test, and continuous variables were analyzed using the Mann-Whitney *U* test. * *p* < 0.05.

**Table 2:** Discontinuation reasons during the whole period.

	Pirfenidone (*n* = 109)	Nintedanib (*n* = 34)	*p* Value
Acute exacerbation	23 (21.1%)	8 (23.5%)	0.819
Disease progression	15 (13.8%)	3 (8.8%)	0.766
Hospital transfer	15 (13.8%)	1 (2.9%)	0.199
Lung cancer	5 (4.6%)	2 (5.9%)	0.674
Adverse effects	34 (31.2%)	24 (70.6%)	0.016 *
Photosensitivity	2 (1.8%)	0 (0.0%)	1.000
Anorexia/ Nausea	16 (14.7%)	6 (17.6%)	0.790
Diarrhea	2 (1.8%)	5 (14.7%)	0.013 *
Liver disorder	0 (0.0%)	9 (26.5%)	<0.001 **
Cardiac disease	1 (0.9%)	0 (0.0%)	1.000
Thrombosis	0 (0.0%)	0 (0.0%)	1.000
Other adverse effects	13 (11.9%)	10 (29.4%)	0.072
Other reasons except the above	9 (8.2%)	0 (0.0%)	0.209
Unknown	11 (10.1%)	2 (5.9%)	0.734

Nominal variables were analyzed using Fisher’s exact test. * *p* < 0.05, ** *p* < 0.01.

## Data Availability

Datasets are freely available upon request.

## Supplementary Materials

The following supporting information can be downloaded at: https://www.mdpi.com/article/10.3390/cells11010143/s1, Figure S1: Comparison of discontinuation reasons during the whole period between pirfenidone and nintedanib.
