# Supplementary material for: Differential Discontinuation Profiles between Pirfenidone and Nintedanib in Patients with Idiopathic Pulmonary Fibrosis
Source: Cells. 2022 Jan 2;11(1):143. doi: 10.3390/cells11010143 (PMC8750555; doi:10.3390/cells11010143)
Supplement: Supplementary file 1 [file cells-11-00143-s001.zip › cells-1496583-supplementary.pdf]

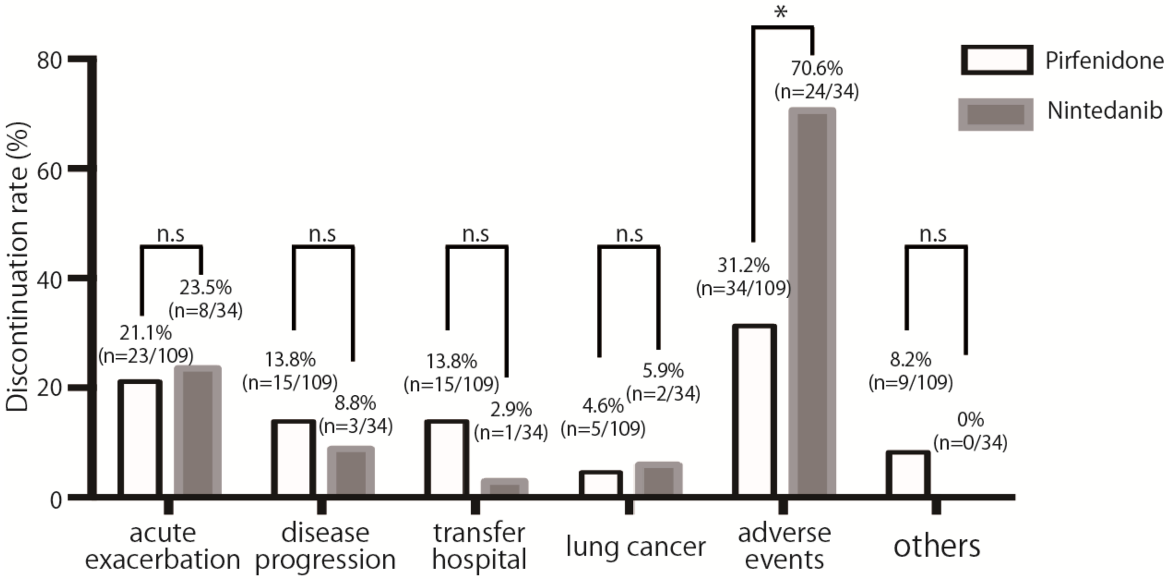

Figure S1. Comparison of discontinuation reasons during the whole period between pirfenidone and nintedanib.
